# Cardiomyocyte Contractile Dysfunction in the APPswe/PS1dE9 Mouse Model of Alzheimer's Disease

**DOI:** 10.1371/journal.pone.0006033

**Published:** 2009-06-24

**Authors:** Subat Turdi, Rui Guo, Anna F. Huff, Eliza M. Wolf, Bruce Culver, Jun Ren

**Affiliations:** Division of Pharmaceutical Sciences & Center for Cardiovascular Research and Alternative Medicine, University of Wyoming College of Health Sciences, Laramie, Wyoming, United States of America; University Mainz, Germany

## Abstract

**Objectives:**

Ample clinical and experimental evidence indicated that patients with Alzheimer's disease display a high incidence of cardiovascular events. This study was designed to examine myocardial histology, cardiomyocyte shortening, intracellular Ca^2+^ homeostasis and regulatory proteins, electrocardiogram, adrenergic response, endoplasmic reticulum (ER) stress and protein carbonyl formation in C57 wild-type (WT) mice and an APPswe/PS1dE9 transgenic (APP/PS1) model for Alzheimer's disease.

**Methods:**

Cardiomyocyte mechanical properties were evaluated including peak shortening (PS), time-to-PS (TPS), time-to-relengthening (TR), maximal velocity of shortening and relengthening (±dL/dt), intracellular Ca^2+^ transient rise and decay.

**Results:**

Little histological changes were observed in APP/PS1 myocardium. Cardiomyocytes from APP/PS1 but not APP or PS1 single mutation mice exhibited depressed PS, reduced±dL/dt, normal TPS and TR compared with WT mice_._ Rise in intracellular Ca^2+^ was lower accompanied by unchanged resting/peak intracellular Ca^2+^ levels and intracellular Ca^2+^ decay in APP/PS1 mice. Cardiomyocytes from APP/PS1 mice exhibited a steeper decline in PS at high frequencies. The responsiveness to adrenergic agonists was dampened although β_1_-adrenergic receptor expression was unchanged in APP/PS1 hearts. Expression of the Ca^2+^ regulatory protein phospholamban and protein carbonyl formation were downregulated and elevated, respectively, associated with unchanged SERCA2a, Na^+^-Ca^2+^ exchanger and ER stress markers in APP/PS1 hearts. Our further study revealed that antioxidant N-acetylcysteine attenuated the contractile dysfunction in APP/PS1 mice.

**Conclusions:**

Our results depicted overt cardiomyocyte mechanical dysfunction in the APP/PS1 Alzheimer's disease model, possibly due to oxidative stress.

## Introduction

Alzheimer's disease (AD) is a devastating neurodegenerative disease leading to memory loss and cognitive impairment. Accumulation of amyloid β-peptide (Aβ) and neurofibrillary tangles are considered hallmarks for neuropathological changes in AD. At this time, neither the precise pathogenesis nor radical cure is available for AD. Although majority of AD cases are idiopathic, an autosomal dominant disorder triggered by mutation in the β-amyloid precursor protein (APP), presenilin 1 (PS1) or presenilin 2 (PS2) has been identified to be responsible for familial AD (FAD) [1]. Interestingly, AD is associated with a high prevalence of heart dysfunction in the elderly . Recent data has indicated a close tie between heart dysfunction and AD. A number of studies have revealed that the onset and progression of AD are closely associated with hypertension, atherosclerosis, diabetes mellitus, hypoxia, myocardial infarction and the presence of ApoE4 allele [2]–[10]. However, limited information is available with regards to the cardiovascular function in AD. Given that the AD genes PS1 and PS2 are also expressed in the heart [11], it may be speculated that mutations in these genes in familial AD affect cardiac function. Presenilins have been suggested to play a key role in the regulation of cardiovascular function. PS1-deficient mice exhibit ventricular septal defect and double outlet right ventricle in the mutant hearts [12]. PS2 also play an important role in cardiac excitation-contraction coupling by interacting with ryanodine receptor type 2 (RyR2) [13]. PS1 and PS2 mutations are associated with a higher risk of dilated cardiomyopathy and heart failure in AD patients [14].

The recent development of transgenic mouse models of AD has provided an important avenue for AD research. Double transgenic mice overexpressing FAD-linked amyloid precursor protein with Swedish mutation and PS-1 with deletion of exon 9 (APPswe/PS1dE9) demonstrated early Aβ deposition as early as 4–6 months of age that correspond to a form of early onset AD [15]. At 9 months of age, APPswe/PS1dE9 transgenic mice display high levels of amyloid β-peptide and numerous amyloid β-peptide deposits [16]. Furthermore, the APPswe/PS1dE9 mice exhibit a consistent β-amyloid accumulation early on and age-associated cognitive decline, consolidating the model for AD [17]. Although many studies have used this transgenic line to investigate aspects of neurodegenerative lesions in AD, no information is available with regards to the cardiac function of this mouse model of AD. Therefore, we took advantage of this mouse model of AD to examine cardiomyocyte mechanical function and underlying mechanisms. Since intracellular Ca^2+^ homeostasis and endoplasmic reticulum (ER) stress are known to be closely associated with cardiomyocyte contractile dysfunction [18], [19], key intracellular Ca^2+^ regulatory proteins such as sarco(endo)plasmic reticulum Ca^2+^-ATPase (SERCA), phospholamban (PLB) and Na^+^-Ca^2+^ exchanger (NCX) as well as crucial protein markers of ER stress including inositol requiring enzyme-1 (IRE-1), calregulin and Bip (GRP78) were also monitored in myocardium of APP/PS1 transgenic and wild-type C57BL/6 mice.

## Methods

### Experimental animals

All animal procedures were conducted in accordance with humane animal care standards outlined in the NIH Guide for the Care and Use of Experimental and were approved the University of Wyoming Animal Care and Use Committee. In brief, 10-month-old male C57BL/6 (WT), APPswe/PS1dE9 (APP/PS1) transgenic mice and mice with transgenic expression of only APPswe (APP) or PS1dE9 (PS1) (Jackson Laboratory, Bar Harbor, Maine, USA) were maintained on a 12:12-h light-dark cycle, with free access to food and water. Production and characterization of the APP/PS1 mouse line were described in detail previously [15]. In brief, APPswe/PS1dE9 mice were developed by co-injection of the two transgene constructs [mouse/human (Mo/Hu) chimeric APP695 harboring the Swedish (K594M/N595L) mutation and exon-9-deleted PS1] into pronuclei with a single genomic insertion site resulting in the two transgenes being transmitted as a single mendelian locus. To identify the transgenic founder mice, genomic DNA was isolated from 1-cm tail clips from 10-month-old mice and genotyped by polymerase chain reaction (PCR) technique using the PCR conditions suggested by Jackson Laboratory. All primers were synthesized by Integrated DNA Technologies Inc. (Coralville, IA, USA) with the following sequences. APPswe: 5′-GAC TGA CCA CTC GAC CAG GTT CTG -3′ and 5′- CTT GTA AGT TGG ATT CTC ATA TCC G-3′; PSEN1dE9: 5′-AAT AGA GAA CGG CAG GAG CA-3′ and 5′-GCC ATG AGG GCA CTA ATC AT-3′). Levels of fasting blood glucose and systolic blood pressure were measured using a glucose monitor (Accu-ChekII, model 792, Boehringer-Mannheim Diagnostics, Indianapolis, IN, USA) and a CODA non-invasive blood pressure system (Kent Scientific Co., Torrington, CT, USA), respectively. At the time of sacrifice, left tibial length was measured with a micrometer.

### Histological examination

Following anesthesia, hearts WT and APP/PSEN mice were excised and immediately placed in 10% neutral-buffered formalin at room temperature for 24 hrs after a brief rinse with PBS. Thereafter, the tissues were dehydrated through serial alcohols and cleared in xylenes. The specimen were then embedded in paraffin, cut in 5 µm sections and stained with hematoxylin and eosin (H&E) or Masson's trichrome (to detect fibrosis) for histological observation [20].

### Morris water maze test

Mice aged 8–10 months from WT and APP/PS1 groups were subjected to behavioral testing. The Morris water maze tests were conducted in a 120-cm (diameter) tank filled with opaque water. The water was kept at 25°C and surrounded by dark walls containing geometric designs that served as distal visual cues. In the first phase of the training, mice were required to find a platform (15×15 cm) marked with a colored pole, placed in different quadrants of the pool. Each animal underwent four trials per day for 4 days, with a maximum of 60 s to find the submerged platform. If the mice failed to find the platform within 60 s, they were physically guided to it and allowed to remain on the platform for 20 s. After visible platform training, two consecutive days of hidden platform training was conducted in which the platform is submerged in the opaque water 1 cm deep, with 4 trials per day. A probe trial was performed at the end of training, in which the platform was removed. Performance in all tasks (latency to platform, time spent in target quadrant) was recorded by a computer-based video tracking system [21].

### ECG measurement

Mice were anesthetized with an intraperitoneal injection of ketamine HCl/xylazine HCl solution (1 ml/kg) and were placed in a supine position on a warm pad (37°C) for acclimatization. Afterwards, three surface probes were inserted subcutaneously and the ECG signal was obtained for 1 minute using a PowerLab (ML866) and Animal Bio Amp (ML136; AD Instruments, Colorado Springs, CO, USA). The signal was analyzed with Chart 5.0 software.

### Cardiomyocyte isolation and in vitro drug treatment

Murine cardiomyocytes were isolated as described [20]. After ketamine/xylazine sedation, hearts were removed and perfused with Ca^2+^ -free Tyrode's solution containing (in mM): NaCl 135, KCl 4.0, MgCl_2_ 1.0, HEPES 10, NaH_2_PO_4_ 0.33, glucose 10, butanedione monoxime 10, and the solution was gassed with 5% CO_2_/95% O_2_. Hearts were digested with Liberase Blendzyme 4 (Hoffmann-La Roche Inc., Indianapolis, IN, USA) for 20 min. Left ventricles were removed and minced before being filtered. Tissue pieces were gently agitated and pellet of cells was resuspended. Extracellular Ca^2+^ was added incrementally back to 1.20 mM over a period of 30 min. Isolated myocytes were used within 8 hrs of isolation. Normally, a yield of 50–60% viable rod-shaped cardiomyocytes with clear sarcomere striations was achieved. Only rod-shaped myocytes with clear edges were selected for mechanical study. To directly assess the role of oxidative stress in AD-associated cardiomyocyte contractile function, a cohort of cardiomyocytes from WT and APP/PS1 mice were incubated at 37°C for 2 hrs in the absence or presence of the antioxidant N-acetylcysteine (NAC, 500 µM) [22] prior to mechanical function assessment.

### Cell shortening/relengthening

Mechanical properties of cardiomyocytes were assessed using a video-based edge-detection system (IonOptix Corporation, Milton, MA, USA) [20]. In brief, cells were placed in a Warner chamber mounted on the stage of an inverted microscope (Olympus, IX-70) with a buffer containing (in mM): 131 NaCl, 4 KCl, 1 CaCl_2_, 1 MgCl_2_, 10 glucose, 10 HEPES, at pH 7.4. The cells were field stimulated with suprathreshold voltage at a frequency of 0.5 Hz, 3 msec duration, using a pair of platinum wires placed on opposite sides of the chamber connected to a FHC stimulator (Brunswick, NE, USA). The polarity of stimulation electrodes was reversed frequently to avoid possible build up of electrolyte by-products. The myocyte being studied was displayed on the computer monitor using an IonOptix My°C am camera, which rapidly scans the image area at every 8.3 msec such that the amplitude and velocity of shortening/relengthening is recorded with good fidelity. The soft-edge software (IonOptix) was used to capture changes in cell length during shortening and relengthening. Cell shortening and relengthening were assessed using the following indices: peak shortening (PS), which indicates peak ventricular contractility; time-to−50% and 90% PS (TPS_50_ and TPS_90_), which indicate rapid and entire phase of myocyte shortening, respectively; time-to−50% and 90% relengthening (TR_50_ and TR_90_), which indicates early and entire phase of myocyte relengthening, respectively; as well as maximal velocity of shortening/relengthening (± dL/dt), which depicts maximal velocity of cardiomyocyte contraction and relaxation. For duration of myocyte shortening and relengthening, 90% rather 100% of the entire duration was used in an effort to avoid noisy signal near baseline. In the case of altering stimulus frequency (from 0.1 Hz to 5.0 Hz), the steady-state contraction of myocyte was achieved (usually after the first five to six beats) before PS amplitude was recorded. The grid-crossing method was used for random cell selection to minimize the influence of heterogeneous cell contraction in response to field stimuli. Myocytes with obvious sarcolemmal blebs or spontaneous contractions were not used for mechanical recording. All measurements were performed at 25–27°C.

### Intracellular Ca^2+^ fluorescence measurement

A separate cohort of myocytes was loaded with fura-2/AM (0.5 µM) for 15 min and fluorescence measurements were recorded with a dual-excitation fluorescence photomultiplier system (Ionoptix) as previously described [20]. In brief, myocytes were placed on an Olympus IX-70 inverted microscope equipped with a temperature-controlled (25°C) Warner chamber and imaged through a Fluor ×40 oil objective. Myocytes were exposed to light emitted by a 75W lamp and passed through either a 360 or a 380 nm filter (bandwidths were±15 nm), while being electrically stimulated to contract at 0.5 Hz. Fluorescence emissions were detected between 480–520 nm by a photomultiplier tube after first illuminating the cardiomyocytes at 360 nm for 500 msec then at 380 nm for the duration of the recording protocol (9 sec at a sampling rate of 333 Hz). The 360 nm excitation scan was repeated for another 500 msec at the end of the protocol and qualitative changes in intracellular Ca^2+^ concentration ([Ca^2+^]i) were inferred from the ratio of the fluorescence intensity at two wavelengths (360/380). This “interpolated” technique was used since 360 nm is the isobestic point for fura-2 at which the numerator is independent of intracellular Ca^2+^ concentration. The majority of the recording was done using the 380 nm filter (except the first and last 500 msec) since the strongest signal is the 380 nm-excited emission. The time course of the fluorescence signal decay was fit to a single exponential equation, and the time constant (τ) was used as a measure of the rate of intracellular Ca^2+^ decay.

### Western blot analysis

The total protein was prepared as described [20]. In brief, tissue samples from mouse ventricles were removed and homogenized in a lysis buffer containing 20 mM Tris (pH 7.4), 150 mM NaCl, 1 mM EDTA, 1 mM EGTA, 1% Triton, 0.1% SDS, and protease inhibitor cocktail. Samples were then sonicated for 15 sec and centrifuged at 13,000× g for 20 min at 4°C. The protein concentration of the supernatant was determined using Protein Assay Reagent (Bio-Rad Laboratories, Richmond, CA, USA). Protein samples were then mixed 1∶2 with Laemmli sample buffer with 5% 2-mercaptoethanol and heated at 95°C for 5 min. Equal amounts (50 µg protein/lane) of the protein mixture, or the SeeBlue Plus2 PreStained markers (Invitrogen, Carlsbad, CA, USA) were separated on 10% or 15% SDS-polyacrylamide gels in a minigel apparatus (Mini-PROTEAN II, Bio-Rad); then were transferred electrophoretically to Nitrocellulose membranes (0.2 µm pore size, Bio-Rad Laboratories, Inc, Hercules, CA, USA). Membranes were incubated for 1 hr at room temperature in a blocking solution containing 5% milk in Tris-buffered saline (TBS). After TBS washed, membranes were incubated overnight at 4°C with primary antibody including rabbit anti-SERCA2a (1∶1,000), rabbit anti-Na^+^-Ca^2+^ exchanger (NCX, 1∶1,000) and mouse anti-phospholamban (PLB, 1∶2,000), rabbit anti-BiP (1∶1,000), goat anti-calregulin (calreticulin) (1∶1,000), rabbit anti-IRE1α (1∶500), rabbit anti-phospho-IRE1α (1∶1,000), rabbit anti-β1 adrenergic receptor antibody (1∶500), rabbit anti-α tubulin (1∶1,000 as loading control) and anti-α smooth muscle actin (1∶1,200 as loading control) antibodies. After three washes with TBS-T to remove excessive primary antibody binding, blots were incubated with horseradish peroxidase (HRP)–conjugated secondary antibody (1∶5,000) for 1 hr at room temperature. The antigens were detected by the luminescence method. Quantification of band density was determined using Quantity One software (Bio-Rad, version 4.4.0) and reported in optical density per square millimeter.

### Protein carbonyl assay

Proteins were extracted and minced to prevent proteolytic degradation. Nucleic acids were eliminated by treating the samples with 1% streptomycin sulphate for 15 min, followed by a 10 min centrifugation (11,000×g). Protein was precipitated by adding an equal volume of 20% trichloroacetic acid (TCA) to protein (0.5 mg) and centrifuged for 1 min. The TCA solution was removed and the sample resuspended in 10 mmol/L 2, 4-dinitrophenylhydrazine (2,4-DNPH) solution. Samples were incubated at room temperature for 15–30 min. After adding 500 µL of 20% TCA, samples were centrifuged for 3 min. The supernatant was discarded, the pellet washed in ethanol: ethyl acetate and allowed to incubate at room temperature for 10 min. The samples were centrifuged again for 3 min and the ethanol: ethyl acetate steps repeated twice or more times. The precipitate was resuspended in 6 mol/L guanidine solution, centrifuged for 3 min and any insoluble debris removed. The maximum absorbance (360–390 nm) of the supernatant was read against appropriate blanks (water, 2 mol/L HCl) and the carbonyl content was calculated using the molar absorption coefficient of 22 000 mol/L^−1^ cm^−1^ [23].

### Statistical analysis

Data are presented as Mean±SEM. Statistical significance (p<0.05) was estimated by ANOVA or t-test, where appropriate.

## Results

### General features of animals and ECG analysis

**Figure 1:**
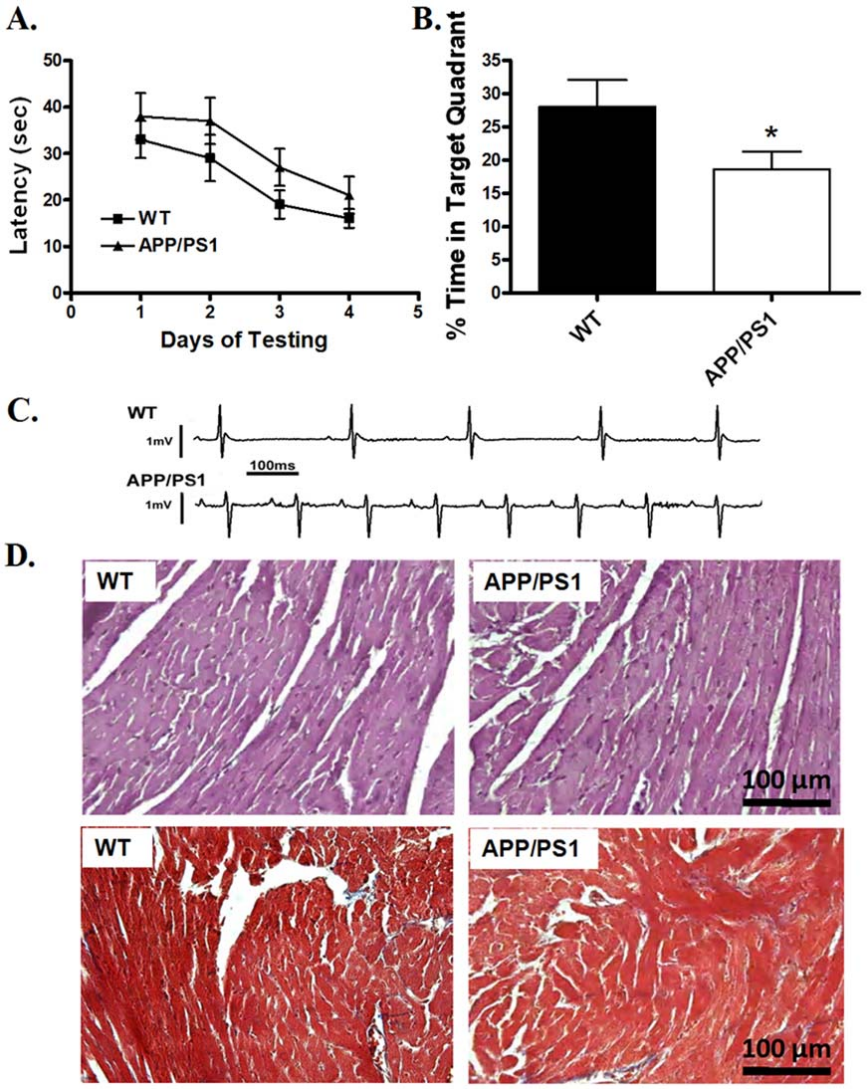
A: The Morris water maze learning curves of mean latency time for WT and APP/PS1 mice; B: Percentage of time spent in the target quadrant for WT and APP/PS1 mice during the probe trial. Data for both panels were obtained from 4 consecutive days of training (4 trials per day); C: Representative ECG traces obtained from WT and APP/PS1 mice; and D: Representative microphotographs of the H&E and Masson's trichrome stained left ventricular tissue sections from WT and APP/PS1 mice (magnification = 400×). Note that Masson's trichrome stains collagen blue to detect interstitial fibrosis. Mean±SEM, n = 5–6 mice for panels A and B, *p<0.05 vs. WT group.

Little difference was identified in tibial length, body and organ (heart, liver and kidney) weights between the APP/PS1 mice and their wild-type (WT) littermates. Both heart-to-body weight and heart weight-to-tibial length ratios were comparable between the two mouse groups. Fasting glucose levels and systolic blood pressure were similar between WT and APP/PS1 mice, excluding the potential contribution of diabetes and hypertension. Surface ECG examination showed that heart rate was significantly faster in the APP/PS1 mice compared to WT mice. Correspondingly, R-R interval was shorter in APP/PS1 mice. P-R interval, Q-T interval and QRS width were all normal in APP/PS1 mice compared with WT. Interestingly, both R wave and QRS complex amplitudes, which are reliable indicatives of myocardial contractility [24], were significantly diminished in APP/PS1 group compared with those from WT control (Table 1). Representative ECG traces from APP/PS1 and WT mice were depicted in Fig. 1.

**Table 1:** General characteristics and ECG properties of WT and APP/PS1) mice.

Mouse group	WT	APP/PS1
Body Weight (g)	27.0±1.0	27.9±1.0
Heart Weight (mg)	163±6	169±6
Heart weight/Body weight (mg/g)	5.51±0.69	5.53±0.69
Tibial length (mm)	16.2±0.9	17.0±0.9
Heart weight /Tibia length (mg/mm)	8.83±0.42	9.19±0.63
Liver Weight (g)	1.58±0.04	1.53±0.04
Kidney Weight (g)	0.42±0.03	0.38±0.03
Fasting Blood Glucose (mg/dl)	110.6±5.0	116.6±10.5
Systolic Blood Pressure (mmHg)	122.9±8.3	126.9±10.2
Heart Rate (bpm)	323±7	382±4^*^
R-R interval (msec)	229±4	164±2^*^
P-R interval (msec)	39.2±5.2	33.6±0.4
Q-T interval (msec)	25.2±0.3	22.1±0.1
QRS Width (msec)	12.6±0.1	10.2±0.6
R Wave Amplitude	0.98±0.22	0.37±0.03^*^
QRS Complex Amplitude	1.12±0.17	0.80±0.02^*^

Mean±SEM, ^*^p<0.05 *vs.* wild-type (WT) group, n = 6–11 mice per group.

### Behavioral evaluation

The standard Morris water maze test was used to evaluate the spatial memory of mice from both groups. Results shown in Fig. 1A indicated that the performance of APP/PS1 mice tended to be poorer (although not significant) than that of WT mice when the platform was hidden. Following 4 days of pre-training, all mice were subjected to a probe trial with the platform taken away. Data from Fig. 1B showed that APP/PS1 mice spent significantly lesser time in the target quadrant than the WT control, indicating spatial memory impairment in the APP/PS1 mice.

### Myocardial histology

To assess the impact of APP/PS1 mutation on myocardial histology, histological studies were performed using the H&E and Masson's trichrome staining techniques. No overt difference was found under the light microscope with regards to the appearance of isolated cardiomyocytes from APP/PS1 and WT groups (data not shown), which was supported H&E and Masson's trichrome staining in myocardial tissue sections from WT and APP/PS1 mice. Little difference was identified for either cardiomyocyte size (H&E staining) or interstitial fibrosis (Masson's trichrome staining) between the two mouse groups (Fig. 1D).

### Cardiomyocyte mechanics in myocytes from APP, PS1 and APP/PS1 transgenic murine model

**Figure 2:**
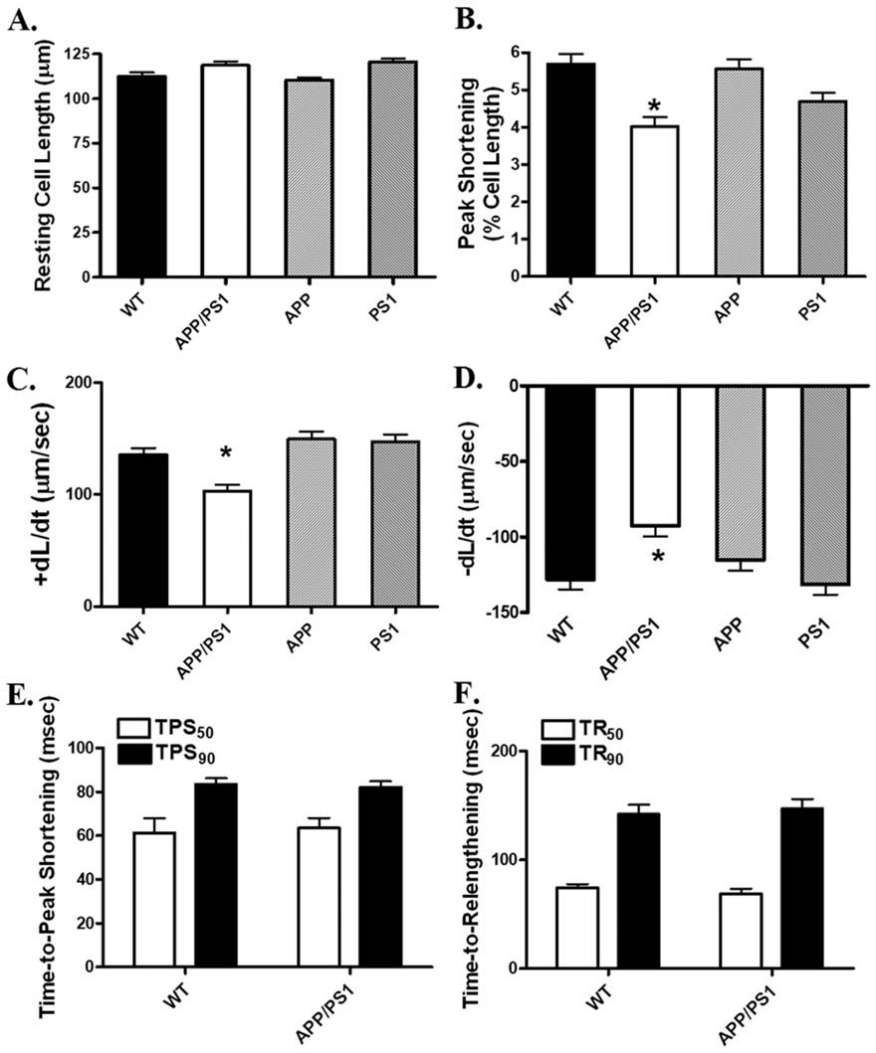
Cardiomyocyte contractile properties in WT, APP/PS1, APP and PS1 mouse hearts. A: Resting cell length (CL); B: Peak shortening (PS, normalized to CL); C: Maximal velocity of shortening (+ dL/dt); D: Maximal velocity of relengthening (- dL/dt); E: Time-to-peak shortening (TPS_50_ and TPS_90_); and F: Time-to-relengthening (TR_50_ and TR_90_). Mean±SEM, n = 132–135 cells from four mice per group, * p<0.05 *vs.* WT group.

The APP/PS1 mouse model used in our study possesses concurrent mutated forms of APP and Presenilin 1 (PS1), both identified separately in human Alzheimer diseases [1]. To examine if individual mutation in APP or PS1 is capable of eliciting myocardial contractile dysfunction independently, cardiomyocyte mechanical function was assessed in the gender- and weight-matched mice with mutation of APP or PS1 (3 mice each, body weight = 27–29 g), or both (APP/PS1). The resting cell lengths (CL) were comparable in WT, APP/PS1, APP and PS1 transgenic mice. At the pacing frequency of 0.5 Hz, peak shortening amplitude (PS) and maximal velocity of shortening/relengthening (±dL/dt) were significantly reduced in cardiomyocytes from APP/PS1 but not APP or PS1 mouse hearts. The time-to-peak shortening (TPS_50_ and TPS_90_) and time-to−90% relengthening (TR_50_ and TR_90_) were comparable between the WT and APP/PS1 mice (Fig. 2). Similarly, TPS_50_, TPS_90_, TR_50_ and TR_90_ were unaffected by single mutation of APP or PS1 at the stimulus frequency of 0.5 Hz (data not shown). Given the higher heart rate in rodents, cell mechanical indices were also examined in WT and APP/PS1 mouse cardiomyocytes paced at 5 Hz. PS and±dL/dt were significantly lowered in APP/PS1 cardiomyocytes (PS: 1.67±0.30%; + dL/dt: 66.4±11.8 µm/sec; - dL/dt: −58.0±13.5 µm/sec, n = 18 cells) compared with those from WT group (PS: 2.88±0.40%; + dL/dt: 91.4±13.8 µm/sec; - dL/dt: −76.5±14.7 µm/sec, n = 21 cells, p<0.05 for all parameters between the two groups). TPS_90_ and TR_90_ were comparable between WT (72±3 and 83±4 msec, respectively) and APP/PS1 (68±4 and 78±5 msec, respectively) mice at the stimulus frequency of 5 Hz.

### Intracellular Ca^2+^ transient properties

**Figure 3:**
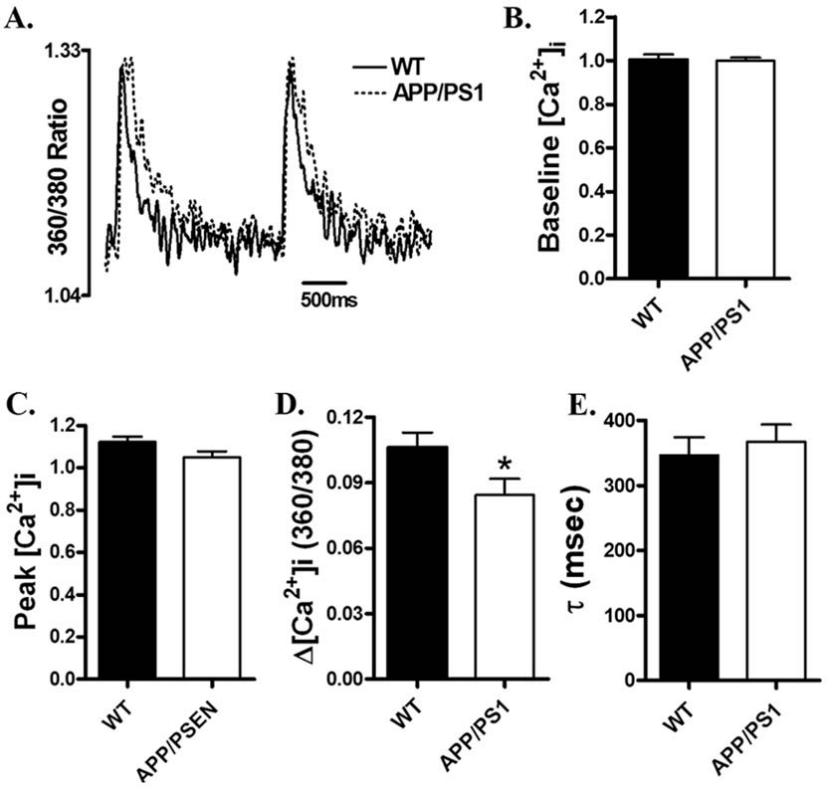
Intracellular Ca^2+^ transient properties in cardiomyocytes from WT and APP/PS1 mouse hearts. A: Representative intracellular Ca^2+^ transients in cardiomyocytes from WT (solid line) and APP/PS1 (dotted line) mice; B: Baseline intracellular Ca^2+^ levels; C: Peak intracellular Ca^2+^ levels; D: Increase of intracellular Ca^2+^ in response to electrical stimuli; and E: Intracellular Ca^2+^ transient decay rate (*τ*). Mean±SEM, n = 62–64 cells from four mice per group, * p<0.05 *vs.* WT group.

To further explore the possible mechanism of action underlying APP/PS1-associated cardiomyocyte mechanical dysfunction, the membrane permeable intracellular Ca^2+^ fluorescent dye fura-2 was used to evaluate intracellular Ca^2+^ homeostasis in cardiomyocytes. Our results depicted unchanged peak and resting intracellular Ca^2+^, as well as intracellular Ca^2+^ decay rate (τ) associated with a slight although significant decrease in intracellular Ca^2+^ rise (Δ[Ca^2+^]i) in cardiomyocytes from APP/PS1 mice compared with those from WT mice (Fig. 3). These data indicated the presence of intracellular Ca^2+^ handling defect in cardiomyocytes from this APP/PS1 transgenic mouse model of AD.

### Effect of increased stimulation frequency on cardiomyocyte shortening

**Figure 4:**
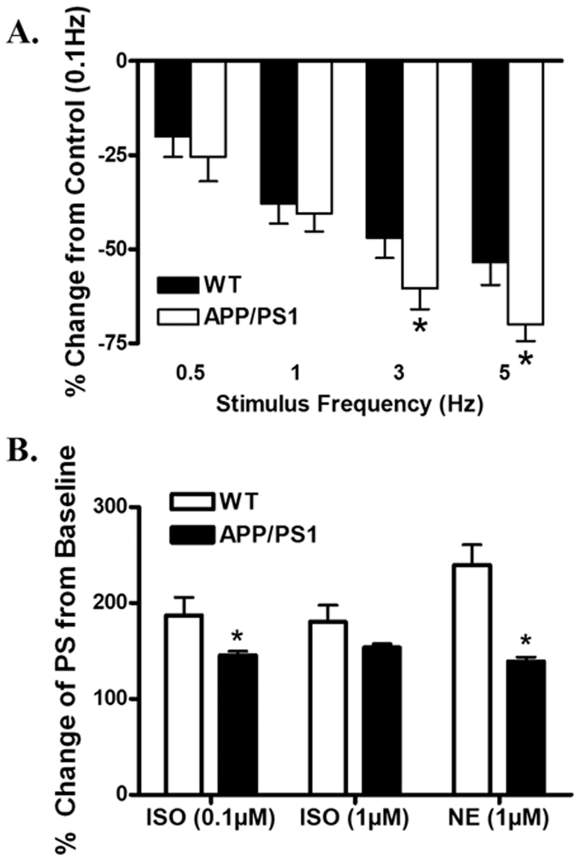
A: Peak shortening amplitude of cardiomyocytes isolated from WT and APP/PS1 mouse hearts at different stimulus frequencies (0.1–5.0 Hz). Peak shortening amplitude is normalized to value obtained at 0.1 Hz of the same cell. B: Adrenergic response in cardiomyocytes from WT and APP/PS1 mice. Data are shown as percent change from baseline in the absence of adrenergic agonists, norepinephrine (NE, 1 µM) or isoproterenol (ISO, 0.1 and 1 µM). Mean±SEM, n = 13–26 cells from four to six mice per data point, * p<0.05 *vs.* WT group.

Mouse hearts normally contract at very high frequencies (400 beat/min), whereas our baseline mechanical assessment was conducted at 0.5 Hz. To investigate the possible role of sarcoplasmic reticulum Ca^2+^ storage and release in APP/PS1 mouse cardiomyocytes, a frequency-response was constructed. Cardiomyocytes were initially stimulated to contract at 0.5 Hz for 5 min to ensure steady-state before commencing the frequency sequence. While increased stimulating frequency (0.1–5.0 Hz) triggered a negative staircase in peak shortening amplitude in both WT and APP/PS1 groups. The degree of decrease was more pronounced in APP/PS1 mice at frequencies greater than 1 Hz (Fig. 4A), indicating existence of compromised SR storage and release of intracellular Ca^2+^ in APP/PS1 mouse hearts.

### Effect of norepinephrine and isoproterenol on cardiomyocyte shortening

To explore the alterations in cardiac excitation-contraction coupling, cardiomyocytes from APP/PS1 and WT mice were exposed to norepinephrine (NE, 1 µM) and isoproterenol (ISO, 0.1 and 1 µM) and myocyte shortening was examined. Results from Fig. 4B displayed a reduced responsiveness to both NE and ISO in cardiomyocytes from APP/PS1 mice compared with those from WT group.

### Protein expression of intracellular Ca^2+^ regulatory proteins, ER stress markers and β-adrenergic receptor

**Figure 5:**
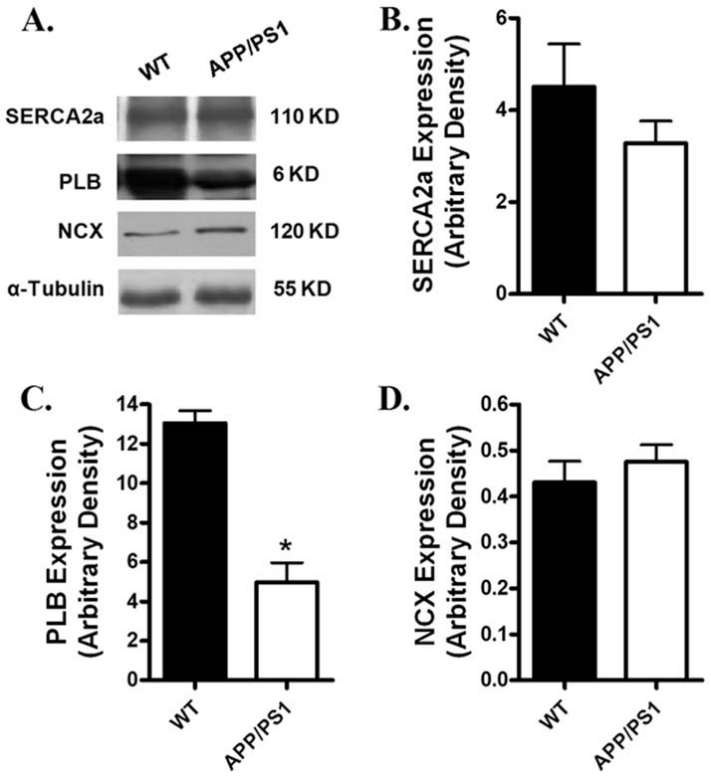
Expression of intracellular Ca^2+^ regulatory proteins in myocardium from WT and APP/PS1 mice. A: Representative gel blots depicting the expression of SERCA2a, NCX and PLB using specific antibodies. α-tubulin was used as an internal loading control; B: SERCA2a; Panel C: Phospholamban (PLB); and D: Na^+^-Ca^2+^ exchanger (NCX). Mean±SEM, n = 4, * p<0.05 *vs.* WT group.

**Figure 6:**
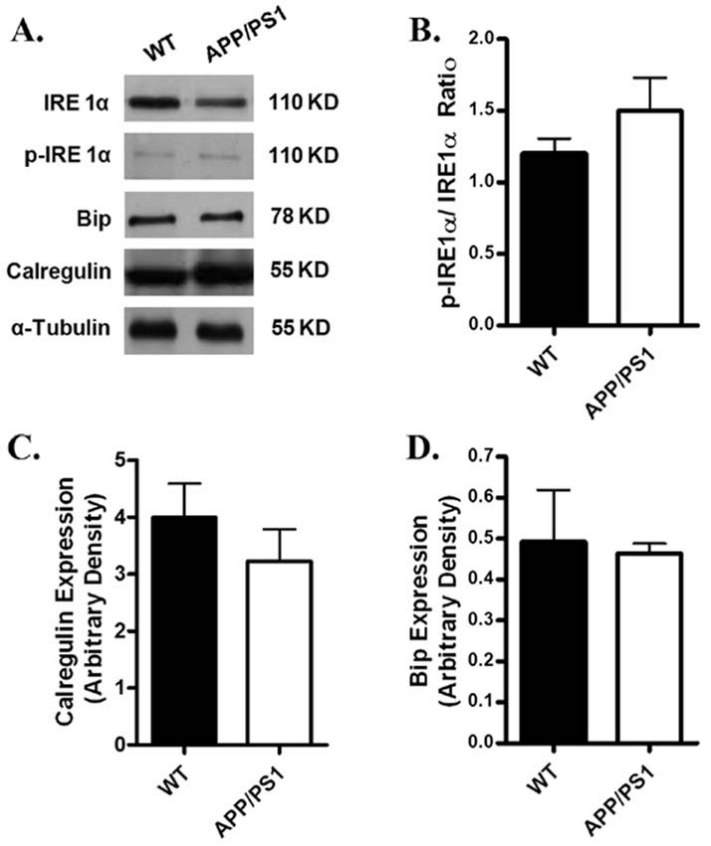
Expression of ER stress markers in myocardium from WT and APP/PS1 mice. A: Representative gel blots depicting the ER stress markers IRE1α, phosphorylated IRE1α, BiP and calregulin (calreticulin) using specific antibodies. α-tubulin was used as an loading control; B: phosphorylated IRE 1α/IRE 1α ratio; C: calregulin; and D: Bip. Mean±SEM, n = 4.

**Figure 7:**
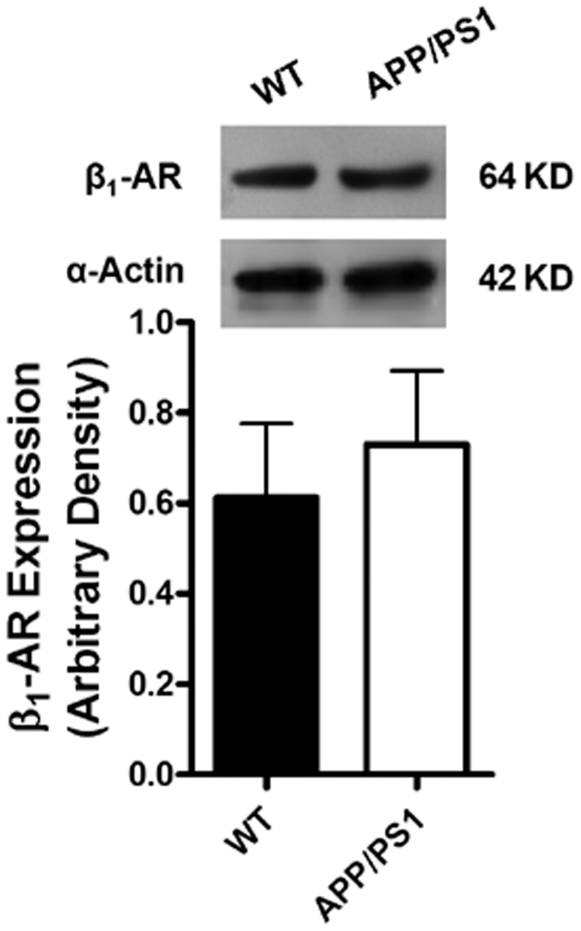
Expression of β1-adrenergic receptor (β1-AR) in myocardium from WT and APP/PS1 mice. Inset: Representative gel blots of β1-AR and α-smooth muscle actin (used as an internal loading control). Mean±SEM, n = 4.

Given that intracellular Ca^2+^ homeostasis is tightly regulated by a cascade of intracellular Ca^2+^ regulatory proteins including SERCA, phospholamban (PLB) and NCX [18], Western blot analysis was performed to evaluate the expression of SERCA2a, PLB and NCX. Our results shown in Fig. 5 revealed a significantly reduced expression of PLB associated with unchanged SERCA2a and NCX in myocardium from APP/PS1 mice compared with the WT group. To explore if alteration in protein trafficking and folding was involved in myocardial dysfunction in APP/PS1 mice, protein expression of the ER stress markers IRE-1α, calregulin and Bip was examined. Results shown in Fig. 6 revealed that IRE-1α, IRE-1α phosphorylation, calregulin and Bip were all comparable between the APP/PS1 and WT groups, not favoring a potential involvement of protein misfolding in APP/PS1 mutation-associated cardiac dysfunction. Our further immunoblotting analysis revealed similar levels of β_1_-adrenergic receptor in myocardium between the APP/PS1 and WT groups (Fig. 7).

### Protein carbonyl formation and effect of antioxidant on cardiomyocyte mechanics in APP/PS1 mice

**Figure 8:**
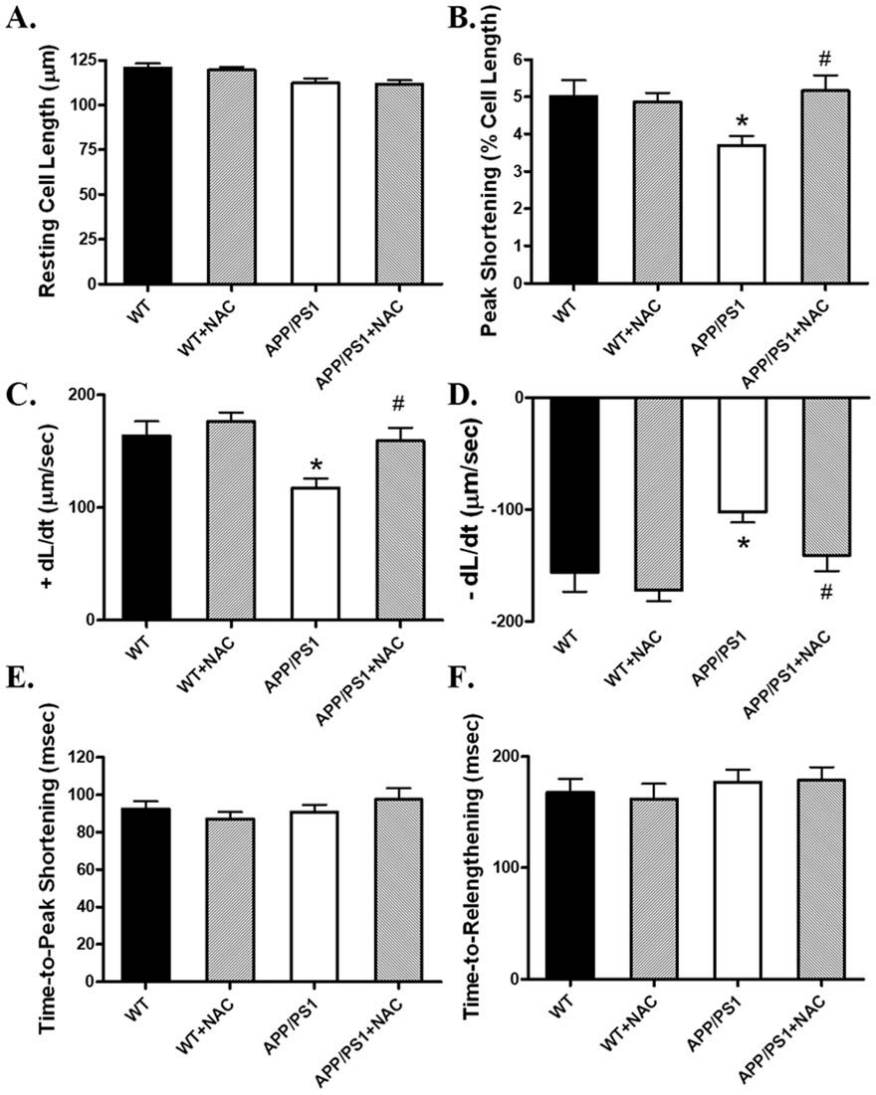
Cardiomyocyte contractile properties in myocytes from WT and APP/PS1 mice incubated for 2 hrs in the presence or absence of the antioxidant N-acetylcysteine (NAC, 500 µM). A: Resting cell length; B: Peak shortening (normalized to cell length); C: Maximal velocity of shortening (+ dL/dt); D: Maximal velocity of relengthening (- dL/dt); E: Time-to-peak shortening (TPS); and F: Time-to−90% relengthening (TR_90_). Mean±SEM, n = 50 cells from 3 mice per group, * p<0.05 *vs.* WT group, # p<0.05 *vs.*APP/PS1 group.

Oxidative stress and subsequent protein oxidation have been reported to play an important role in the pathogenesis of AD brain [25]–[28]. To assess the cardiac oxidative protein damage in the APP/PS1 AD model, protein carbonyl formation was determined. Results from the present study revealed that myocardial protein carbonyl formation was significantly higher in APP/PS1 (3.139±0.716 nmol/mg protein) than WT mice (0.993±0.215 nmol/mg protein, p<0.05 between the two groups). To further examine the role of oxidative stress in APP/PS1-induced cardiac contractile defects, freshly isolated cardiomyocytes from WT and APP/PS1 mice were incubated for 2 hrs in the absence or presence of the antioxidant NAC (500 µM). Our data revealed that NAC effectively ablated APP/PS1-induced mechanical defects (depressed PS and±dL/dt) without eliciting any effects on cardiomyocyte mechanics itself (Fig. 8). These data provided direct evidence for a likely role of oxidative stress in AD-induced cardiac contractile dysfunction, consistent with elevated protein carbonyl formation.

## Discussion

Data from our current study have clearly depicted cardiomyocyte contractile dysfunction in the APPswe/PS1dE9 (APP/PS1) transgenic model of AD. The major mechanical defects observed in cardiomyocytes from APP/PS1 mice include decreased cell shortening, reduced velocities of shortening/relengthening at the stimulus of both 0.5 Hz and 5 Hz and exaggerated frequency response. Our results indicated that individual mutation in either APP or PS1 alone is insufficient to trigger overtly compromised cardiomyocyte contractile function compared with concurrent presence of both. These findings are consistent with literature that the expression of APPswe alone results in less Aβ production than APPswe/PS1dE9 and PS1dE9 transgene enhances the production of Aβ in APPswe mice while little effect exerted by itself [29], [30]. Our further study revealed the possible contribution of decreased adrenergic response, dysregulated intracellular Ca^2+^ homeostasis, protein damage and oxidative stress to impaired cardiomyocyte mechanical function in APP/PS1 mice.

In our study, the APP/PS1 mice were euglycemic and normotensive, excluding potential contribution of diabetes and hypertension to the compromised cardiac function. Our data revealed overt mechanical changes associated with exacerbated spatial learning and memory impairment in APP/PS1 mice. Approximately 2% of populations in developed countries are afflicted with AD [31]. In 2004, AD was ranked the 7th among the 10 leading causes of death in the US [32]. Although there has been an intensive effort to understand AD, the main focus thus far is to elucidate the pathogenesis of neurodegeneration in AD brain with rather limited data for the organ-specific complications of AD. During the past two decades, a growing body of evidence has revealed many risk factors of cardiac diseases which are also detrimental to AD, indicating a close link between cardiac diseases and AD [33]. To the best of our knowledge, our current study is the first report to provide direct evidence of cardiac dysfunction in AD using this transgenic line.

In our study, the heart rate was higher in AD mice. This may be associated with cardiac autonomic dysfunction in AD although conflicting data were seen. Elevated catecholamine levels and ablated vagal tone were reported in AD patients [34], [35], supporting the higher heart rate observed in our current study. Impaired vagal parasympathetic function was reported in AD patients evaluated by R-R interval variation (RRIV) and sympathetic skin response and orthostatic cardiovascular reflexes [36]. In addition, QT dispersion was found to be tightly correlated with the degree of cognitive impairment in AD patients. As shown in our current study, surface ECG revealed for the first time increased heart rate, reduced RR interval and diminished QRS amplitude in the APP/PS1 mice compared with WT mice.

The most prominent cardiac dysfunctions in the APP/PS1 AD mouse model observed in our study were reduced cardiomyocyte contraction and diminished maximal velocity of contraction/relaxation (±dL/dt) at both low and high stimulus frequencies associated with unchanged duration of contraction and relaxation. Several factors may contribute to the impaired mechanical function including defected contractile proteins (e.g., actin, myosin heavy chain isoform), reduced intracellular Ca^2+^ release, depressed SR Ca^2+^ load and altered myofilament Ca^2+^ sensitivity. The intracellular Ca^2+^ measurement confirmed a decrease in intracellular Ca^2+^ rise associated with unchanged resting and peak intracellular Ca^2+^ levels in cardiomyocytes from APP/PS1 mice. The unchanged intracellular Ca^2+^ decay coincides with the normal TR_50_ and TR_90_ in APP/PS1 mouse cardiomyocytes, which is also supported by the normal protein levels of SERCA2a and NCX, the main machineries to remove Ca^2+^ from cytosolic space for cardiac relaxation to occur, in APP/PS1 myocardium. In addition, our data revealed exaggerated decline in the steady-state myocyte peak shortening in response to increased stimulating frequency in APP/PS1 mice, suggesting a possibly reduced capacity in SR Ca^2+^ storage and release in this murine model of AD. The reduced cardiomyocyte shortening capacity received convincing support from the reduced R-wave height in ECG reading. Our data revealed down-regulation of phospholamban, an endogenous inhibitor of SERCA, in APP/PS1 mouse hearts. Although the link between reduced phospholamban expression and altered cardiac contractile function in APP/PS1 hearts is unclear, reduced phospholamban levels may trigger altered intracellular Ca^2+^ homeostasis *en route* to affect cardiomyocyte contractile function [18]. Our study revealed enhanced protein carbonyl formation in the absence of ER stress in myocardium from APP/PS1 mice, suggesting a likely role of oxidative stress in the altered cardiomyocyte contractile function in APP/PS1 mice. This notion received further support from our *in vitro* study where antioxidant NAC alleviates the cardiomyocyte mechanical dysfunction in APP/PS1 mice.

Catecholamine desensitization is a hallmark of heart failure [37]. It has been shown that heart failure may trigger catecholamine desensitization to both isoproterenol and norepinephrine in the presence and absence of ganglionic blockade [37]. This is in line with our observation of reduced responsiveness to norepinephrine and isoproterenol in APP/PS1 mouse cardiomyocytes. However, the lack of overt change in myocardial histology and β_1_-adrenergic receptor expression suggest the absence of any advanced heart failure and adrenergic desensitization in APP/PS1 hearts. Although sympathetic denervation may lead to postsynaptic supersensitivity due to lack of neural uptake for norepinephrine [37], to what extent adrenergic denervation or desensitization contributes to myocardial complications in AD patients remains controversial. Several reports have been seen to discriminate changes in cardiac sympathetic innervation of AD patients from other neurodegenerative disease such as dementia with Lewy bodies and vascular dementia [38]–[40]. These studies measured cardiac uptake of metaiodobenzylguanidine (MIBG), a physiologic analogue of norepinephrine [41], as a mean of noninvasive index of the integrity of sympathetic neurotransmission in the heart of AD patients. It was found that cardiac MIBG uptake in AD patients was essentially unaffected compared with other neourodegenrative diseases. Nevertheless, subtle differences may exist in the diagnostic criteria of AD and different stages of disease process.

In summary, this study provides evidence for the first time of altered cardiomyocyte contractile function in the APP/PS1 transgenic model of AD. Cardiomyocytes from APP/PS1 mice exhibited decreased inotropic response and impaired intracellular Ca^2+^ homeostasis possibly due to mechanisms associated with reduced intracellular Ca^2+^ release, altered expression in Ca^2+^ regulatory proteins, reduced adrenergic function and oxidative damage. However, other mechanisms should not be excluded at this time such as susceptibility of myofilament Ca^2+^ sensitivity and altered expression of cardiac contractile proteins. Future study is warranted to elucidate the subcellular signaling mechanisms responsible for cardiac abnormalities in AD.
